# Altered spontaneous calcium signaling of *in situ* chondrocytes in human osteoarthritic cartilage

**DOI:** 10.1038/s41598-017-17172-w

**Published:** 2017-12-06

**Authors:** Xiaoyuan Gong, Wenbin Xie, Bin Wang, Lingchuan Gu, Fuyou Wang, Xiang Ren, Cheng Chen, Liu Yang

**Affiliations:** 1 Center for Joint Surgery, Southwest Hospital, Third Military Medical University, Chongqing, 400038 P.R. China; 2 Department of Joint Surgery, First Affiliated Hospital, Hunan Normal University, Changsha, Hunan 410005 P.R. China; 3 Institute of Life Sciences, Chongqing Medical University, Chongqing, 400016 P.R. China

## Abstract

Intracellular calcium ([Ca^2+^]_i_) signaling is an essential universal secondary messenger in articular chondrocytes. However, little is known about its spatiotemporal features in the context of osteoarthritis (OA). Herein, by examining the cartilage samples collected from patients undergoing knee arthroscopic surgery, we investigated the spatiotemporal features of spontaneous [Ca^2+^]_i_ signaling in *in situ* chondrocytes at different OA stages. Our data showed zonal dependent spontaneous [Ca^2+^]_i_ signaling in healthy cartilage samples under 4 mM calcium environment. This signal was significantly attenuated in healthy cartilage samples but increased in early-degenerated cartilage when cultured in 0 mM calcium environment. No significant difference was found in [Ca^2+^]_i_ intensity oscillation in chondrocytes located in middle zones among ICRS 1–3 samples under both 4 and 0 mM calcium environments. However, the correlation was found in deep zone chondrocytes incubated in 4 mM calcium environment. In addition, increased protein abundance of Ca_v_3.3 T-type voltage dependent calcium channel and Nfatc2 activity were observed in early-degenerated cartilage samples. The present study exhibited OA severity dependent spatiotemporal features of spontaneous [Ca^2+^]_i_ oscillations of *in situ* chondrocytes, which might reflect the zonal specific role of chondrocytes during OA progression and provide new insight in articular cartilage degradation during OA progression.

## Introduction

Osteoarthritis (OA), which involves the dysfunction of adult articular cartilage, is the most common form of joint disease with manifestations of damaged articular cartilage, and may result in arthralgia, joint deformation, and limited mobility in patients. OA is the second leading cause of long-term disability in adults. Several risk factors of OA (*e.g*. genetics, age, and mechanical loading) have been identified^1^; however, the mechanisms of OA initiation and progression are yet to be understood. Chondrocytes, as the highly specialized, metabolically active cells, regulate the development, maintenance, and repair of extracellular matrix (ECM) in articular cartilage^2^. Unlike its low cellular activity and matrix turnover ability in healthy articular cartilage^3,4^, chondrocytes play a pivotal role in OA initiation and progression by upregulating expression of matrix metalloproteinases (MMP-1, MMP-14), aggrecanase (ADAMTS-5) and inflammatory cytokines (interleukin-1α/β, and tumor necrosis factor -α)^4,5^.

Intracellular calcium ([Ca^2+^]_i_) signaling is an essential universal secondary messenger that mediates the cellular metabolic activity in chondrocytes^6–10^. Based on its spatiotemporal parameters (amplitude, frequency, and duty cycle), encoded information is deciphered by various downstream transcription factors (*e.g*. nuclear factor of activated T cells (Nfat), nuclear factor-κB (NF-κB), c-Jun N-terminal kinase 1 (JNK1), myocyte enhancer factor-2 (MEF2), and cAMP response element-binding protein (CREB)), and ultimately leads to a range of metabolic and signaling processes^11^. Due to its unique role in extracellular-intracellular signal transduction, [Ca^2+^]_i_ signaling has been widely studied in articular cartilage. It has been shown that chondrocytes not only respond to mechanical and chemical stimuli with [Ca^2+^]_i_ signaling via modulating extracellular calcium influx and intracellular calcium release^6–10^, but also reflect its peri-cellular environment (*e.g*. osmotic stress, growth factors, and cytokines) with repetitive spontaneous [Ca^2+^]_i_ signaling when statically cultured^6,12^, which plays a crucial role in regulating ECM metabolic activity^12^. With abnormal joint physiology such as increased mechanical loading and surrounding inflammatory activity, response of chondrocytes to their peri-cellular environment may be significantly altered, potentially contributing to the onset or progression of OA.

Articular cartilage is a highly organized tissue, its zonal differences in biochemical content and bioelectrical properties are directly associated with depth-dependent changes in mechanical properties^13,14^. Rolauffs and colleagues demonstrated the significantly increased cell density in the deep zone of synchronized primary metabolic chondrons of articular cartilage, compared with superficial zone^15^. Several studies^16,17^ confirmed the variety in cytokine sensitivity of chondrocytes located in different zones, indicating the distinctive role of chondrocytes located in different zones during the pathological process of OA. Previous studies investigating chondrocyte [Ca^2+^]_i_ signaling were mainly focused on cells removed from their physiological environment, such as 2D cultures or chondrocytes/gel constructs^6,18^. These studies neglected the potential role of calcium channel activity linked to ECM, as well as the specialized peri-cellular matrix (PCM) that affected chondrocyte mechanics^19^.

In the present study, we aimed to investigate the spontaneous [Ca^2+^]_i_ signaling of human articular chondrocytes located in its native PCM and ECM, and compared the zonal differences in [Ca^2+^]_i_ signaling of *in situ* human chondrocytes. By comparing the spatiotemporal features of spontaneous [Ca^2+^]_i_ signaling in *in situ* chondrocytes at different OA stages, the correlation between spontaneous [Ca^2+^]_i_ signaling and OA severity was investigated. In addition, to address the possible mechanism of OA chondrocytes [Ca^2+^]_i_ signaling alternation and downstream regulation of [Ca^2+^]_i_ signaling, the protein level of T-type voltage-dependent calcium channels (VDCCs) and Nfatc2 activity were analyzed using total knee arthroplasty (TKA) samples.

## Results

### Histology evaluation

**Figure 1:**
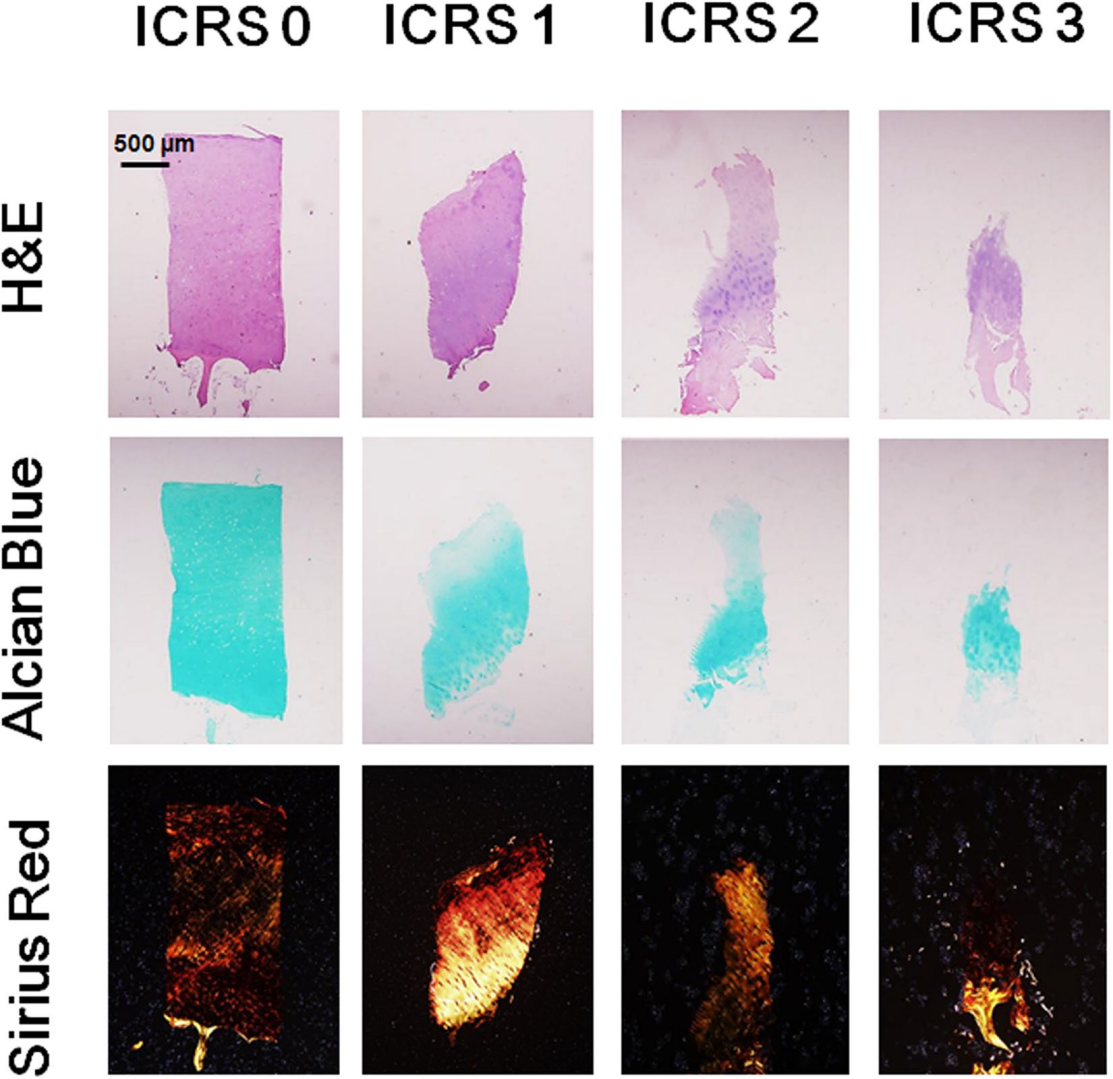
Histological feature of ICRS 1–3 grades articular cartilage samples collected from patients undergoing knee arthroscopic surgery. From top to bottom, the severity of collected articular cartilage samples was evaluated by hematoxylin and eosin (H&E), alcian blue, and sirius red, respectively (n = 3–4 for each ICRS grade).

Based on the International Cartilage Research Society (ICRS) grading system, the severity of each collected human articular cartilage sample was verified by histomorphometry approaches. Later on, the spatiotemporal features of spontaneous [Ca^2+^]_i_ signaling analyzed from each sample were classified into ICRS grades. Representative hematoxylin and eosin (H&E), alcian blue, and sirius red staining images for ICRS 0–3 grades were shown in Fig. 1. As expected, cartilage samples showed increased wear from top to bottom. As shown by alcian blue staining, gradually decreased proteoglycan concentration was most pronounced at superficial and deep zones in degenerated samples (ICRS 1–3). Sirius red staining suggested dramatically changed collagen composition from type П to type І at ICRS 1 samples, and absence of both types of collagen fibrils at late stage OA samples (ICRS 3).

### Propagation of calcium signaling in middle and deep zones of articular cartilage samples

**Figure 2:**
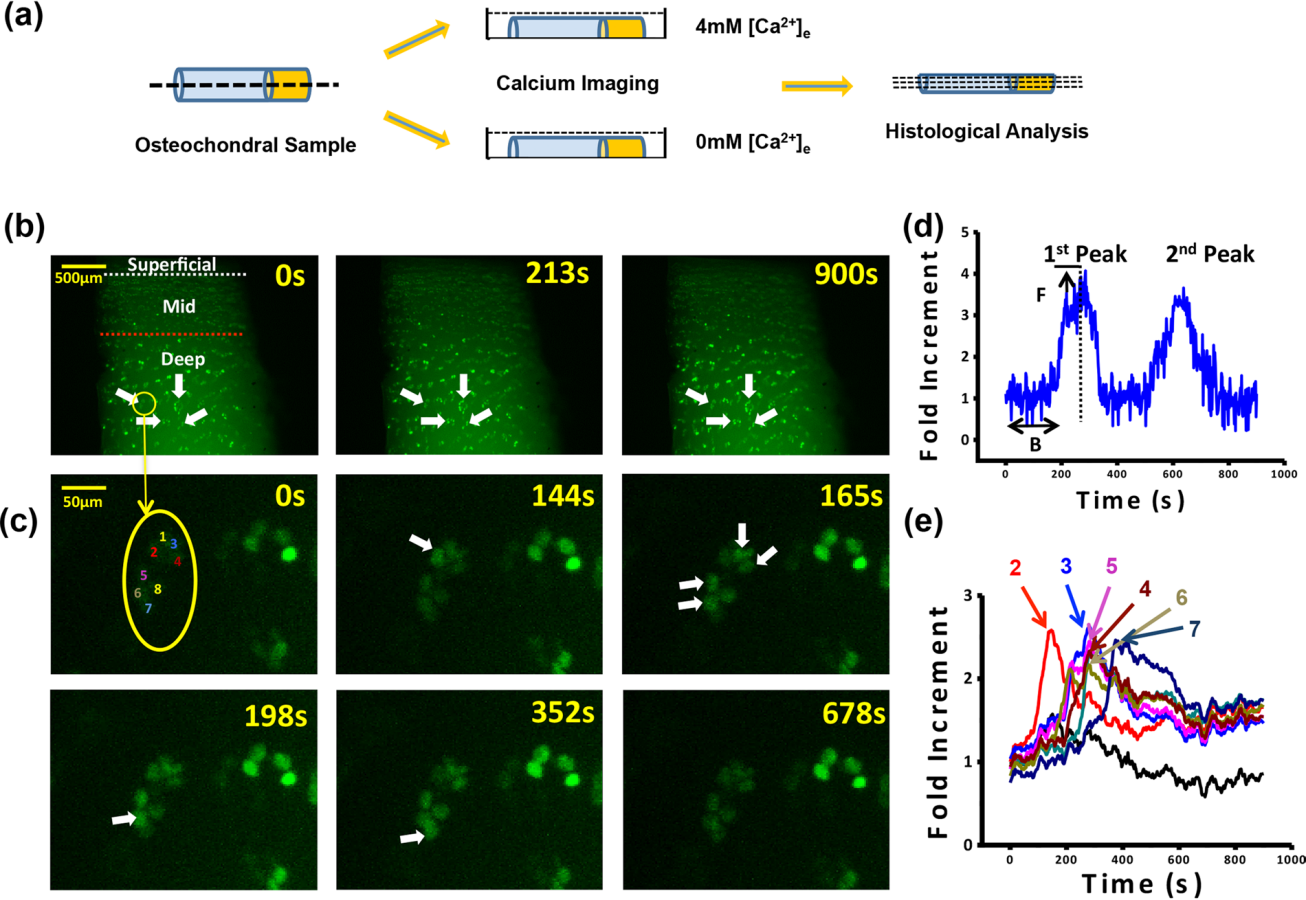
Real-time calcium imaging of *in situ* human chondrocytes. (**a**) Study design, 2 mm in diameter cylindrical osteochondral samples at different ICRS grades were collected form femoral condyle during arthroscopy. Samples were cut into two half-cylinders and used for calcium imaging at 4 mM and 0 mM [Ca^2+^]_e_ environments. To classify calcium imaging data, histology evaluation was performed on these samples afterward. (**b**) Typical [Ca^2+^]_i_ image series in ICRS 0 articular cartilage sample and in (**c**) single lacuna of the same sample. (**d**) A typical [Ca^2+^]_i_ intensity oscillations curve of a chondrocyte and the definitions of spatiotemporal parameters. (**e**) Typical [Ca^2+^]_i_ intensity oscillations curve in the same lacuna shown in (**c**) (white arrows indicated cells displayed spontaneous [Ca^2+^]_i_ signal).

Spontaneous calcium signaling was observed in chondrocytes within both normal (ICRS 0) and OA (ICRS 1–3) articular cartilages without extraneous stimuli. Typical [Ca^2+^]_i_ oscillations in ICRS 0 cartilage sample were shown in Fig. 2b. Propagation of [Ca^2+^]_i_ oscillations were observed in a large portion of chondrons in middle and deep zones of both normal and OA cartilages. As shown in Fig. 2c and e, [Ca^2+^]_i_ oscillations propagated from cell number 2 to neighboring cells (cell number 3, 4, 5 and 6), and from cell number 6 to cell number 7. The time lag of [Ca^2+^]_i_ peaks between each propagation of cell 1–7 also suggested this phenomenon.

### Zonal difference of spontaneous calcium signaling in normal chondrocytes

**Figure 3:**
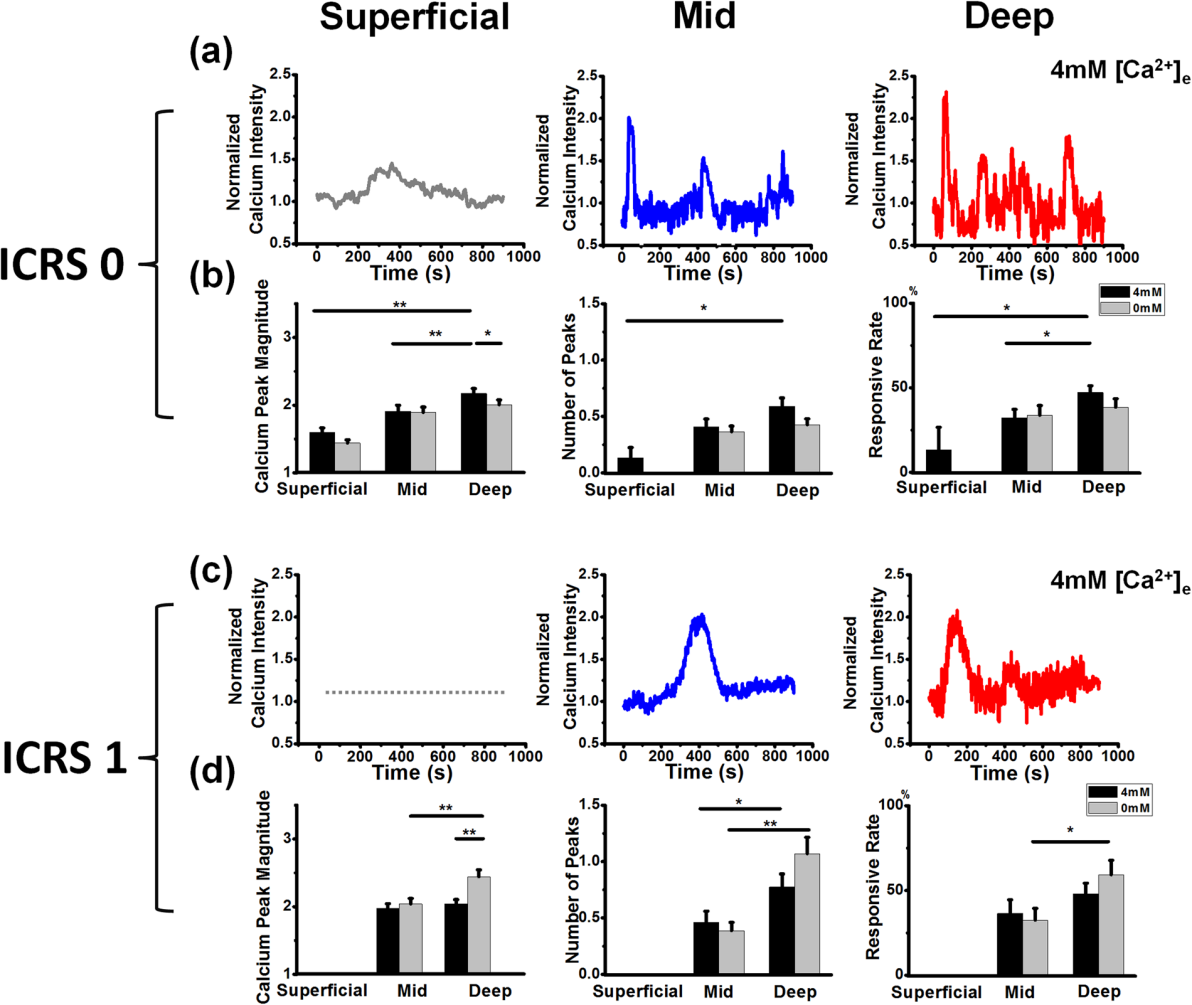
Characterization of spatiotemporal features of spontaneous [Ca^2+^]_i_ signaling in chondrocyte of health (ICSR 0) and early OA samples (ICRS 1). Representative [Ca^2+^]_i_ oscillation curves of chondrocytes located in superficial, middle, and deep zones of (**a**) ICRS 0 and (**c**) ICRS 1 sample, spatiotemporal features (calcium peak magnitude, number of peaks, and responsive rate) of spontaneous [Ca^2+^]_i_ signaling in chondrocyte located in different zones of (**b**) ICRS 0 and (**d**) ICRS 1 samples when cultured in either 0 mM or 4 mM [Ca^2+^]_e_ environments. Results were expressed as mean ± SEM (for calcium peak magnitude and number of peaks, 15–102 cells were analyzed; for responsive rate, 4 individual samples were analyzed in superficial zones, and 10–20 chondrons were analyzed in middle and deep zones, ^*^ *P* < 0.05; ^**^ *P* < 0.01).

Spatiotemporal features of [Ca^2+^]_i_ signaling in *in situ* chondrocytes were zonal, [Ca^2+^]_e_ concentration, and ICRS grade dependent. Typical [Ca^2+^]_i_ oscillations for both normal (ICRS 0) and early (ICRS 1) OA cartilages under 4 mM [Ca^2+^]_e_ environment were shown in Fig. 3. In ICRS 0 samples, [Ca^2+^]_i_ oscillations increased with the depth of cartilage (Fig. 3a). During the 15-min recording period, only a small portion of chondrocytes (13.33 ± 13.33%) located in the superficial zone displayed [Ca^2+^]_i_ peaks larger than 2 fold increment (calcium peak magnitude: 1.60 ± 0.07) (Fig. 3b), while a significantly larger portion of chondrocytes (47.17 ± 4.01%, *P* < 0.05) located in single chondrons of deep zone demonstrated repetitive (0.60 ± 0.07 vs. 0.13 ± 0.09 peaks, *P* < 0.05) and stronger [Ca^2+^]_i_ peaks (calcium peak magnitude: 2.17 ± 0.08, *P* < 0.01) during the same recording period (Fig. 3b). Compared with chondrocytes in deep zone, chondrocytes located in middle zone showed moderate [Ca^2+^]_i_ oscillations: 32.25 ± 5.00% (*P* < 0.05) cells showed valid [Ca^2+^]_i_ peak, with 0.41 ± 0.07 peaks (*P* > 0.05) and calcium peak magnitude of 1.91 ± 0.09 fold (*P* < 0.01) during the same recording period.

In 0 mM [Ca^2+^]_e_ environment, the difference in each parameter of [Ca^2+^]_i_ oscillations between middle and deep zones was abolished (*P* > 0.05), due to decreased [Ca^2+^]_i_ oscillations of chondrocytes in deep zone when cultured in 0 mM [Ca^2+^]_e_ environment (calcium peak magnitude: 2.17 ± 0.08 vs. 2.00 ± 0.07, *P* < 0.05). Furthermore, [Ca^2+^]_i_ oscillations in chondrocytes in the superficial zone were completely diminished when cartilage was incubated in 0 mM [Ca^2+^]_e_ environment.

### Altered spontaneous calcium signaling pattern in early OA chondrocytes

In early OA (ICRS 1) cartilage, zonal difference in [Ca^2+^]_i_ oscillations of chondrocytes between middle and deep zones was attenuated under 4 mM [Ca^2+^]_e_ environment (calcium peak magnitude: 1.98 ± 0.07 vs. 2.04 ± 0.07, *P* > 0.05, number of peaks: 0.46 ± 0.10 vs. 0.78 ± 0.12, *P* < 0.05, responsive rate: 36.50 ± 8.03% vs. 47.95 ± 6.41%, *P* > 0.05) (Fig. 3d). However, when ICRS 1 samples were incubated in 0 mM [Ca^2+^]_e_ environment, chondrocytes in deep zone displayed enhanced [Ca^2+^]_i_ oscillations compared to chondrocytes in middle zone (calcium peak magnitude: 2.44 ± 0.10 vs. 2.04 ± 0.08, *P* < 0.01, number of peaks: 1.07 ± 0.15 vs. 0.39 ± 0.07, *P* < 0.01, responsive rate: 59.29 ± 8.54% vs. 32.50 ± 6.97%, *P* < 0.05). Furthermore, compared with 4 mM [Ca^2+^]_e_ culture, chondrocytes in ICRS 1 deep zone displayed stronger [Ca^2+^]_i_ when incubated in 0 mM [Ca^2+^]_e_ environment (calcium peak magnitude: 2.44 ± 0.10 vs. 2.04 ± 0.07, *P* < 0.01).

### Comparison of spontaneous calcium signaling of chondrocytes in different ICRS grade samples

**Figure 4:**
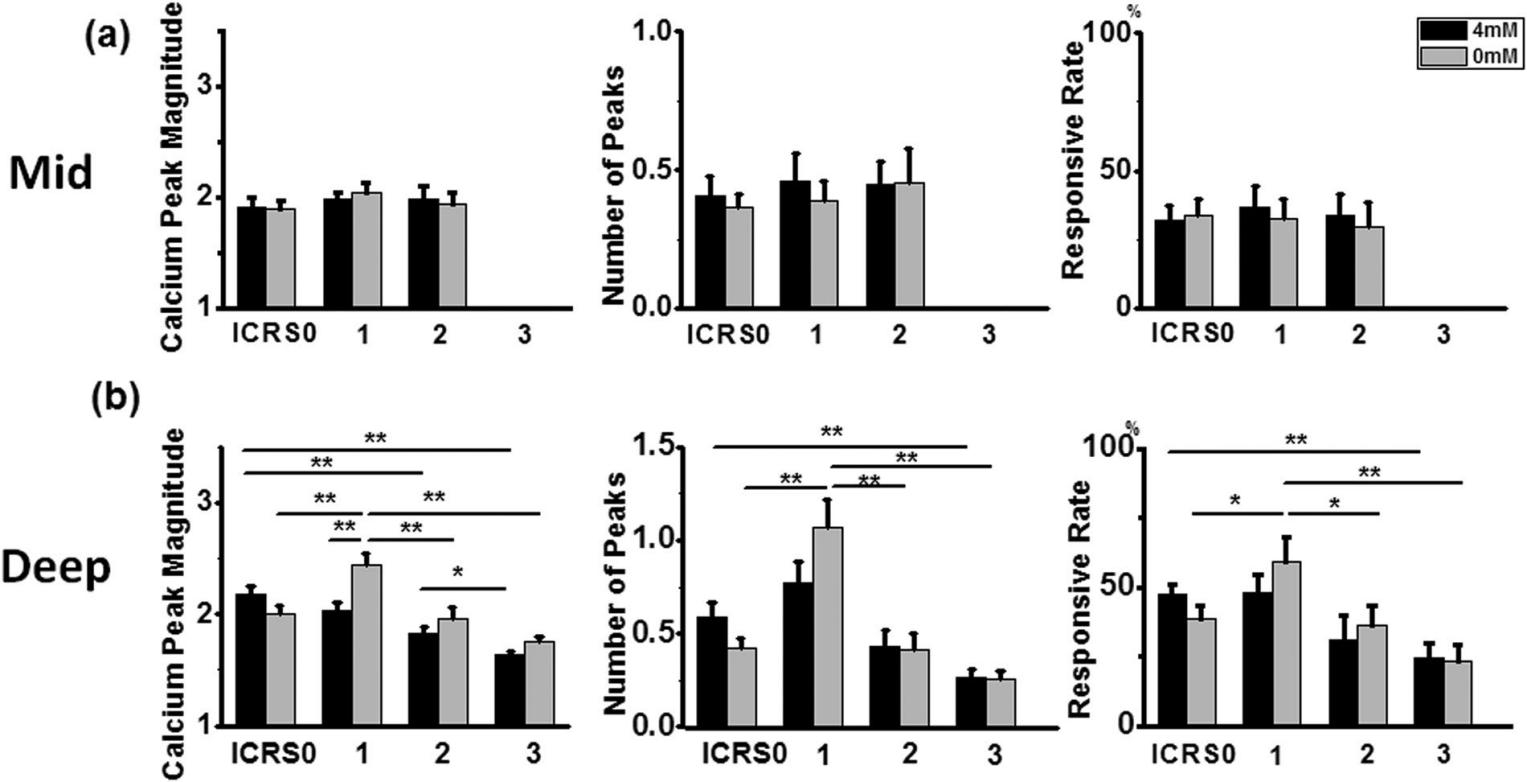
Comparison of spatiotemporal features of spontaneous [Ca^2+^]_i_ signaling among ICRS 0–3 samples. (**a**) No significant difference was found in [Ca^2+^]_i_ oscillation in chondrocytes located in middle zones under either 4 mM or 0 mM [Ca^2+^]_e_ environment. (**b**) Decreased [Ca^2+^]_i_ signaling intensity was found in deep zone chondrocytes of degenerated cartilage when incubated in 4 mM calcium environment. When samples were incubated in calcium 0 mM [Ca^2+^]_e_ environment, ICRS 1 sample showed strongest [Ca^2+^]_i_ oscillation. Results were expressed as mean ± SEM (for calcium peak magnitude and number of peaks, 46–107 cells were analyzed; for responsive rate, 10–20 chondrons were analyzed in middle and deep zones, ^*^ *P* < 0.05; ^**^ *P* < 0.01).

Comparison of spatiotemporal features of [Ca^2+^]_i_ signaling was not performed in superficial zones since this region was only presented in ICRS 0 samples. No zonal or [Ca^2+^]_e_ concentration-dependent difference was found in the middle zones of all ICRS grades (Fig. 4). In contrast, chondrocytes in deep zone showed significant variation in [Ca^2+^]_i_ oscillations. The gradually decreased intensity of spontaneous calcium signaling in ICRS 0, 2, and 3 samples were observed. Compared with ICRS 0 samples, chondrocytes in ICRS 2 (calcium peak magnitude: 1.83 ± 0.06 vs. 2.17 ± 0.08, *P* < 0.01) and ICRS 3 (calcium peak magnitude: 1.64 ± 0.04 vs. 2.17 ± 0.08, *P* < 0.01, number of peaks: 0.26 ± 0.04 vs. 0.60 ± 0.07, *P* < 0.01, responsive rate: 24.65 ± 5.14% vs. 47.17 ± 4.01%, *P* < 0.01) samples demonstrated significantly weaker [Ca^2+^]_i_ oscillations when incubated in 4 mM [Ca^2+^]_e_ environment. Also, chondrocytes in ICRS 3 samples showed even weaker [Ca^2+^]_i_ oscillations than that displayed by cells in ICRS 2 samples (calcium peak magnitude: 1.64 ± 0.04 vs. 1.83 ± 0.06, *P* < 0.05). However, this trend was not found in ICRS 1 samples. Chondrocytes located in the deep zone of ICRS 1 samples showed comparable [Ca^2+^]_i_ oscillations of ICRS 0 chondrocytes when incubated in 4 mM [Ca^2+^]_e_ environment (Fig. 4b, black bars).

In 0 mM [Ca^2+^]_e_ environment, the difference in [Ca^2+^]_i_ oscillations among ICRS grades was attenuated, except for ICRS 1 samples. Compared with other grades, chondrocytes located in deep zone of ICRS 1 samples showed the strongest [Ca^2+^]_i_ oscillations (calcium peak magnitude: 2.44 ± 0.10, *P* < 0.01, number of peaks: 1.07 ± 0.15, *P* < 0.01, responsive rate: 59.29 ± 8.54%, *P* < 0.05 compared with ICRS 0 and 2, and <0.01 compared with ICRS3, respectively). Furthermore, compared with samples within each ICRS grade in 4 mM [Ca^2+^]_e_ environment, no significant difference was found in each ICRS 0, 2, 3 samples; while in ICRS 1 samples, significantly stronger [Ca^2+^]_i_ oscillations were found in 0 mM [Ca^2+^]_e_ environment as described above.

### Correlation between spontaneous calcium signaling and OA severity

To analyze the possible correlation between spontaneous calcium signaling and OA severity, Spearman’s rank correlation coefficient method was used to analyze the relationship of [Ca^2+^]_i_ signaling parameters of ICRS 0–3 samples to ICRS grading system under 4 mM and 0 mM [Ca^2+^]_e_ environment, respectively (Table 1). Results suggested that calcium peak magnitude (r = −0.33, *P* < 0.001) and responsive rate (r = −0.486, *P* < 0.001) were moderately correlated with ICRS grade when cartilage samples were incubated in 4 mM [Ca^2+^]_e_ environment. In contrast, when cartilage samples were incubated in 0 mM [Ca^2+^]_e_ environment, the correlation between parameters of [Ca^2+^]_i_ signaling and ICRS grading system was very weak.

**Table 1:** Spearman rank correlation coefficient (r) between the parameters of [Ca^2+^]_i_ signaling in deep zones and ICRS grades.

	ICRS grade		Sample size
	r	*p*	
Calcium peak magnitude (4 mM)	−0.330	<0.001	342 cells
Calcium peak magnitude (0 mM)	−0.124	0.023	331 cells
Number of peaks (4 mM)	−0.205	<0.001	358 cells
Number of peaks (0 mM)	−0.151	0.006	330 cells
Responsive rate (4 mM)	−0.486	<0.001	70 chondrons
Responsive rate (0 mM)	−0.219	0.093	65 chondrons

### Increased protein abundance of T-type VDCCs in early-degenerated articular cartilage

**Figure 5:**
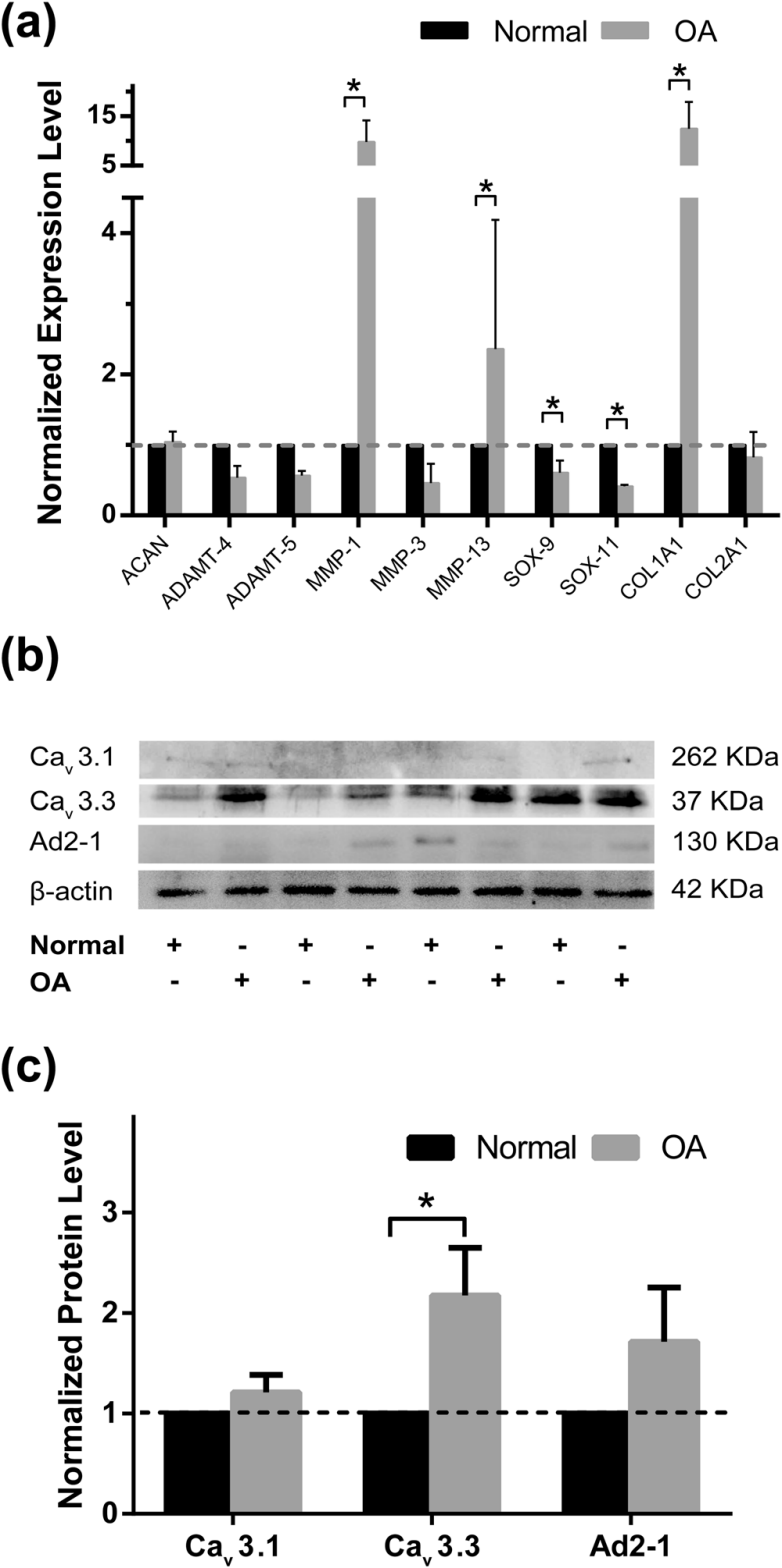
Expression level of chondrogenic genes and protein abundance of T-VDCCs in non-degenerated and early-degenerated articular cartilage samples. (**a**) Compared with non-degenerated cartilage (Normal, black bars), chondrocytes located in early-degenerated area (OA, grey bars) of TKA samples showed increased expression level of MMP-1, MMP-13, and COL1A1; and decreased SOX-9 and SOX-11 (^*^ *P* < 0.05) (n = 6). (**b**) Representative Western blot detection of Ca_v_3.1, Ca_v_3.3, A2D-1, and β-actin (full-length blots/gels were presented in Supplementary Figure S1). (**c**) Compared with non-degenerated cartilage (Normal, black bars), significantly increased protein level of Ca_v_3.3 was noticed in early-degenerated (OA, grey bars) regions (^*^ *P* < 0.05) (n = 8).

To explore the mechanism of unregulated [Ca^2+^]_i_ signaling in ICRS 1 samples, non-degenerated (Normal) and early-degenerated (OA) regions of TKA cartilage samples were used for RT-qPCR and Western boltting analyses. Compared with non-degenerated cartilage, chondrocytes in early-degenerated cartilage had significantly higher level of MMP-1, MMP-13, and COL1A1; and lower level of SOX-9 and SOX-11 (Fig. 5a). Western boltting data suggested that chondrocytes located in both regions expressed T-type Ca_v_3.1, Ca_v_3.3, and α2σ1 proteins, but negatively expressed Ca_v_3.2 protein. When compared with the non-degenerated region, significantly increased protein level of Ca_v_3.3 was noticed in the early-degenerated region (Fig. 5b and c).

### Increased Nfatc2 activity in early-degenerated articular cartilage

**Figure 6:**
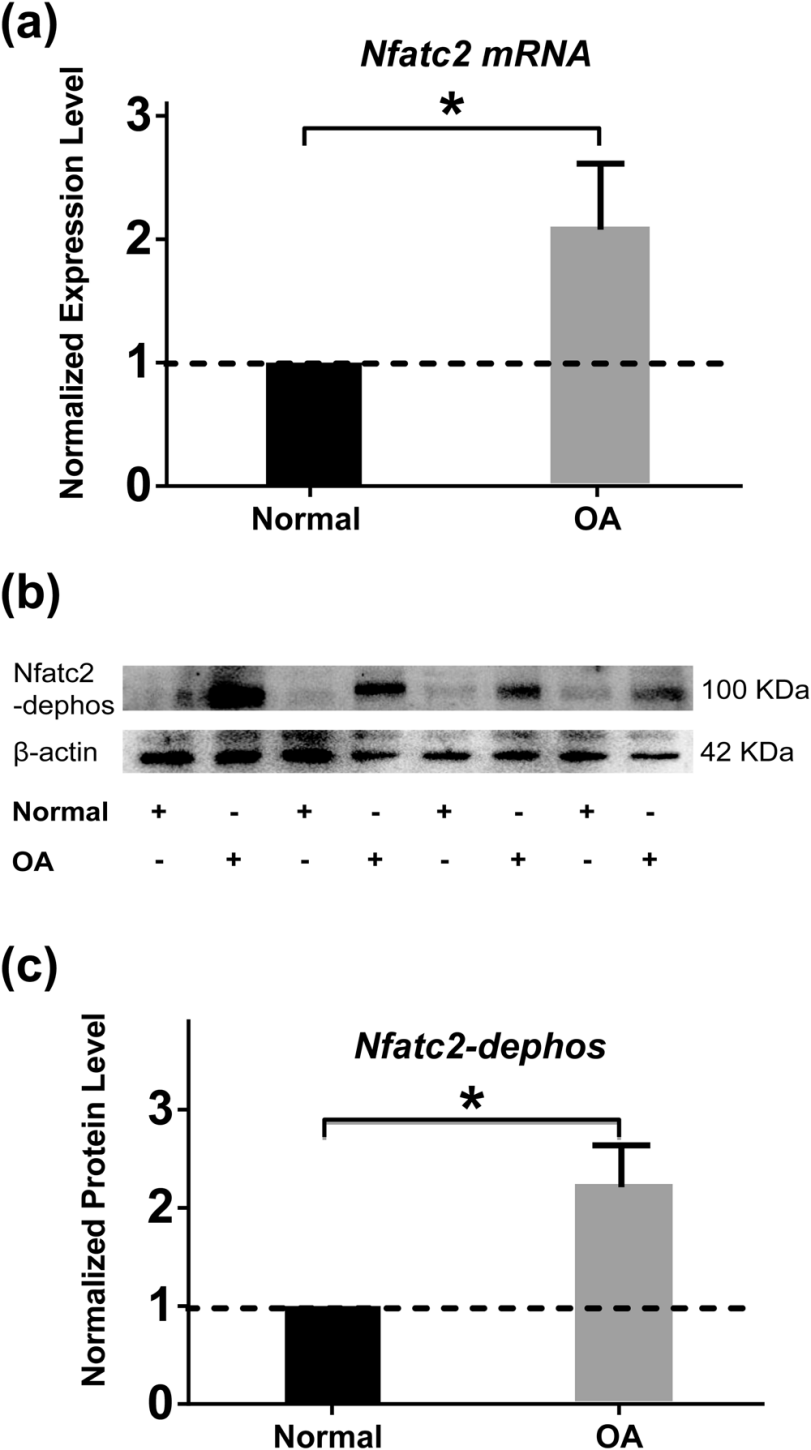
Expression level and activity of Nfactc2 in non-degenerated and early-degenerated articular cartilage sample. (**a**) Compared with non-degenerated cartilage (Normal, black bars), RT-qPCR data indicated that chondrocytes located in the early-degenerated area (OA, grey bars) of TKA sample showed increased expression level of Nfatc2 (^*^ *P* < 0.05) (n = 6). (**b**) Representative Western blot detection of de-phosphorylated Nfatc2 (full-length blots/gels are presented in Supplementary Figure S1). (**c**) Compared with non-degenerated cartilage (Normal, black bars), Significantly increased dephosphorylated Nfatc2 was found in early-degenerated (OA, grey bars) regions (^*^ *P* < 0.05) (n = 8).

To explore the role of unregulated [Ca^2+^]_i_ signaling in OA progression, the activity of Nfatc2, downstream transcription factors of [Ca^2+^]_i_ signaling was analyzed using RT-qPCR and Western boltting in TKA cartilage samples. Results indicated significantly up-regulated Nfatc2 expression (Fig. 6a), and increased protein level of de-phosphorylated Nfatc2 in chondrocytes located in early-degenerated regions (Fig. 6b and c).

## Discussion

The objective of this study was to investigate the spatiotemporal features of spontaneous [Ca^2+^]_i_ signaling in *in situ* chondrocytes of human healthy and OA articular cartilage, which might shed light on understanding the molecular mechanism of articular cartilage degradation in OA. Our data showed a significant difference of extracellular calcium dependent spontaneous [Ca^2+^]_i_ signaling between zones in healthy cartilage, while this zonal difference was not observed in degenerated cartilage. In early-degenerated cartilage, abnormal [Ca^2+^]_i_ activities in deep zone were seen when samples were cultured in both 0 mM and 4 mM [Ca^2+^]_e_ environment. Increased Ca_v_3.3 protein abundance and Nfatc2 activity were possibly associated with abnormal [Ca^2+^]_i_ activities in early-degenerated cartilage. Also, the intensity of spontaneous [Ca^2+^]_i_ signaling was found negatively correlated with OA severity. These data indicated that the zonal specific role of chondrocytes in maintaining ECM integrity in human healthy articular cartilages and reflected alternation of chondrocytes metabolic activity during OA progression.

It is recently discovered that both isolated and *in situ* animal chondrocytes can display repetitive [Ca^2+^]_i_ signaling without any extracellular stimuli. Occasional spontaneous [Ca^2+^]_i_ signaling in rabbit chondrocytes located in native ECM and 2D culture was first observed by Kono *et al*., in which spontaneous [Ca^2+^]_i_ signaling did not propagate to adjacent cells unless mechanically stimulated^6^. This observation was later demonstrated by O’Conor *et al*., who showed that ~20% of 3D cultured porcine chondrocytes displayed spontaneous [Ca^2+^]_i_ signaling^7^. More recently, Zhou *et al*. showed that ~45% of *in situ* bovine mature chondrocytes displayed spontaneous [Ca^2+^]_i_ signaling, which had a significantly higher responsive rate than that of chondrocytes located in the middle zone of juvenile cartilage samples^20^. This phenomenon was validated in human chondrocytes in the present study, in which ~32.25% of chondrocytes displayed spontaneous [Ca^2+^]_i_ signaling in the middle zone of healthy articular cartilage samples. However, the exact mechanism of spontaneous [Ca^2+^]_i_ signaling in chondrocytes remains unclear. Similar to excitable cells such as neurons and myocytes^21^, mature chondrocytes are well equipped with rich complement of calcium channels such as voltage-dependent calcium channels (VDCCs)^22^, mechanical sensitive calcium channels (MSCCs)^18^, transient receptor potential vanilloid (TRPV)^7^, and Ca^2+^ release-activated Ca^2+^ (CRAC) channel^23^. These calcium channels synergistically regulate [Ca^2+^]_i_ homeostasis by modulating extracellular calcium influx^24^ and intracellular calcium release from endoplasmic reticulum^25^. In the present study, total abolishment and significant attenuation of spontaneous [Ca^2+^]_i_ signaling in chondrocytes located in superficial and deep zones were noticed when incubated in 0 mM [Ca^2+^]_e_ environment. Since cells in these zones were located in the peripheral area of cartilage samples and more susceptible to extracellular changes of environmental calcium concentration than cells in the middle zones, our results suggested that similar to MSCs^26^, spontaneous [Ca^2+^]_i_ signaling in chondrocytes was dependent on extracellular calcium influx. However, unlike the results presented by Kono *et al*., our observation suggested that [Ca^2+^]_i_ signal propagation existed in most chondrons of both healthy and OA cartilage. This contradiction could be explained by variation in the area where the cartilage sample was examined. In our observation, propagation of [Ca^2+^]_i_ signal was only observed in middle and deep zones, where 5–10 chondrocytes were closely located in one chondron. Since articular cartilage is an avascular and an eurogenic connective tissue, in which a chondrocyte is isolated by its native PCM and lacks direct cell-cell connection^27^; [Ca^2+^]_i_ signaling at a distance^12^ is possibly through extracellular messengers such as nitric oxide (NO) and nucleotides and nucleosides, ATP, uridine triphosphate (UTP), or adenosine diphosphate (ADP)^28^. Propagated [Ca^2+^]_i_ signal in *in situ* chondrocytes may serve as an efficient intercellular communication pathway^6^.

Similar to mechanically induced [Ca^2+^]_i_ response, spontaneous [Ca^2+^]_i_ signaling has been shown to regulate ECM maintenance in cartilage. Zhou *et al*. recently suggested that sustained spontaneous [Ca^2+^]_i_ signaling enhanced mechanical properties of cartilage samples during long-term *ex-vivo* culture^12^. By analyzing the spatiotemporal features of spontaneous [Ca^2+^]_i_ signaling in *in situ* chondrocytes of calf articular cartilage, they showed a high correlation between responsive rate and mechanical property of cultured explants. This observation was confirmed in the present study, in which we showed an increased responsive rate of spontaneous [Ca^2+^]_i_ signaling from superficial to deep zone in normal cartilage. This phenomenon might reflect the distinct function of chondrocytes located in different zones revealed by microarray analysis^29^, which demonstrated that chondrocytes isolated from deep zones synthesized more proteoglycans than cells from the superficial zone^30^. In addition, Spearman’s rank correlation coefficient method also revealed that the responsive rate of spontaneous [Ca^2+^]_i_ signaling was most correlated with the severity of cartilage degradation when incubated in 4 mM [Ca^2+^]_e_ environment, suggesting that the spontaneous [Ca^2+^]_i_ signaling could serve as a useful indicator of OA progression.

When spontaneous [Ca^2+^]_i_ signaling of *in situ* chondrocytes was compared among different ICRS grade samples, no significant difference was seen in middle zone chondrocytes, indicating that chondrocytes in this zone were relatively inactive during the whole process of OA. In contrast, decreased intensity of [Ca^2+^]_i_ signaling in deep zones of late-stage OA cartilages was found among grades. Decreased calcium channel activity caused by alternation in channel structure of OA chondrocytes could be one of the reasons^22^. Animal model using combination of orally administered monosodium iodoacetate (MIA) and treadmill over-loading showed decreased protein level of TRPV5, a voltage-independent cation channel in OA chondrocytes^31^. A recent study using OA animal model and human sample also revealed decreased TRPV6 expression in degraded cartilages^32^. In addition, by using RNA-seq analysis, Dunn *et al*. compared gene expression changes in human OA cartilage samples and found that α2/δ, a subunit of voltage-dependent calcium channels regulating channel properties and increasing the functional channel^33^, was significantly down-regulated^34^. By using the larger volume of cartilage sample acquired during total knee arthroplasty (TKA) surgery, we showed that chondrocytes in the early-degenerated area had significantly increased expression level of MMP-1, MMP-13, COL1A1; and decreased expression level of SOX-9 and SOX-11. In addition, increased protein abundance of Ca_v_3.3, a α1 subunit of T-type VDCC in the early-degenerated area was noticed. The transiently active Ca_v_3.3 T-type VDCC requires weak membrane depolarization for activation. Increased Ca_v_3.3 protein abundance might decrease the membrane potential^35^ and pronounce [Ca^2+^]_i_ signaling of chondrocytes in deep zone of early OA sample when few calcium irons were presented; and ultimately activated Nfatc2, a transcription factor has been shown to be crucial in chondrogenesis in tracheal cartilage^36^, and function regulation of adult chondrocytes^37^. Despite the fact that early OA was most pronounced in the superficial and middle zones^38^, our data suggested a unique role of deep zone chondrocytes in the initiation of OA. In early OA, the increased Nfatc2 activity might serve as a compensatory mechanism to withstand the established damage in articular cartilage. There is a growing consensus that OA is a whole-joint disease^39^. Increased turnover of subchondral bone seen in OA patients^40^ and animal models^41^ indicated an important role of subchondral bone-deep zone cartilage crosstalk during the onset of OA. Our previous studies using human OA samples and destabilization of medial meniscus (DMM) model showed decreased thickness in calcified cartilage zone (CCZ) at early stage^40^, accompanied by increased invading vessels density in this region^41^. These observations suggested elevated paracrine transport of inflammatory factors and cytokines released from subchondral bone could influence the calcium channel activities of deep zone chondrocytes, ultimately altering the ECM components such as proteoglycan concentration and composition of collagen observed in the present study.

The present study has several limitations. Firstly, although human biopsy samples at different OA stages represent the real progression of OA and retain the native PCM and ECM, they also suffer from disadvantages such as small sample volume, limited sample quantity, and restricted location for sample collection. In the current study, only a small volume of cartilage sample was acquired from each patient to prevent further damage to articular cartilage. To exclude the individual variation between patients, larger samples size and paired control sample representing non-degenerated cartilage from the same individual seems necessary for mechanism study. Secondly, the role of elevated spontaneous [Ca^2+^]_i_ signaling in OA progression remains unclear. More stable sample source such as cartilage collected from the large-animal model is needed for performing [Ca^2+^]_i_ dependent downstream pathway analysis.

Despite the limitations, the current study presented the direct evidence that *in situ* chondrocytes resided in both healthy and OA human articular cartilage displayed spontaneous [Ca^2+^]_i_ signaling, and indicated that the spatiotemporal features of the [Ca^2+^]_i_ oscillations were both zonal and OA severity dependent. While the exact mechanism needs further investigation, the changes of [Ca^2+^]_i_ signaling might reflect the zonal specific role of chondrocytes during OA progression and provide new insight into molecular mechanism of articular cartilage degradation during OA progression. Also, our observation here also suggested that spontaneous [Ca^2+^]_i_ signaling of *in situ* chondrocytes could serve as a potential biomarker for clinical diagnosis of early OA.

## Methods

### Cartilage explants

Articular cartilage samples for calcium imaging and histology evaluation were collected from patients receiving knee arthroscopy by one senior surgeon in Center for Joint Surgery, Southwest Hospital, Third Military Medical University, Chongqing, China. Informed consent was obtained from participant patients. During the surgical process, a single 2 mm in diameter cylindrical osteochondral sample was collected using a custom-designed biopsy needle that fits the arthroscopic system. For ICRS 0 group, healthy cartilage samples were collected in the non-load bearing area in knee joints with no sign of cartilage degeneration. For ICRS 1–3 groups, samples representing the most severe wear in each knee were collected from the femoral condyle before chondroplasty. The severity of cartilage degradation in their knee joints was evaluated during surgery based on ICRS Visual Assessment Scale, and confirmed by histomorphometry approaches afterward. Cartilage samples containing defects caused by trauma, tumor, or rheumatoid arthritis (RA) were excluded from this study. 3–4 cartilage samples for each ICRS grade were collected from 15 patients (Age: 42.69 ± 2.5 years; BMI: 23.55 ± 0.64; 5 males, 10 females; Table 2). This study was conducted in accordance with the declaration of Helsinki and with approval from the Ethics Committee of the Southwest Hospital of Third Military Medical University (Chongqing, China).

**Table 2:** Demographic Data of Patients^a^.

ICRS 0				ICRS 1				ICRS 2				ICRS 3			
Sample set	Age	Gender	BMI	Sample set	Age	Gender	BMI	Sample set	Age	Gender	BMI	Sample set	Age	Gender	BMI
1	28	M	25.5	1	46	F	22.6	1	42	F	27.1	1	53	F	28.6
2	38	F	20.9	2	44	M	22	2	49	F	24.8	2	37	F	24.2
3	24	M	22.5	3	50	F	23.8	3	45	M	23.4	3	44	M	23
4	50	F	18.5	4	—	—		4	51	F	23.4	4	40	F	22.9
Mean	35	—	21.85		46.67		22.8		46.75		24.68		43.5		24.68

^a^Values were presented as mean ± SEM. No statistical differences were found for age and BMI among each group (*P* > 0.05).

### Calcium imaging of *in-situ* chondrocytes

Harvested samples containing full-thickness cartilage and subchondral bone were immediately transferred to laboratory in PBS (Hyclone, Beijing, China). Samples were carefully cut into 2 half-cylinders using a scalpel. [Ca^2+^]_i_ transients were monitored in *in situ* chondrocytes using Fluo-8 AM (AAT Bioquest, CA, USA) and fluorescence microscopy (IX71, Olympus, Tokyo, Japan). As shown in Fig. 2, collected samples were stained by 5 µM Fluo-8 AM at 37 °C for 30 min; excess dye was removed by washing with PBS for three times. Stained cartilage samples were then transferred into 96-well plates and kept in standard bath solution contained (in mM): NaCl, 144; NaH_2_PO_4_, 0.33; KCl, 4.0; MgCl_2_, 0.53; glucose, 5.5; HEPES, 5.0; CaCl_2_, 0 or 4 mM (the pH was adjusted to 7.4 with NaOH) at 37 °C for 15 min to remove any agitation during staining process^26^. Fluorescent images of chondrocytes were then recorded using 4 x objectives at room temperature for 15 min while the sample was undisturbed.

### Image processing

To analyze the [Ca^2+^]_i_ signaling, recorded image sequences were analyzed using Image J (Version 1.44p). The cartilage samples were separated into superficial, middle and deep zones in the image software according to chondrocyte/chondron size and arrangement. In detail, parallelly-arranged cells in cartilage surface were considered as superficial zone chondrocytes; cells in middle range of cartilage samples, and with the formation of medium-size chondrons in random arrangement were considered as middle zone chondrocytes; hypertrophy cells in deep range, and with the formation of large-size chondrons in vertical arrangement were considered as deep zone chondrocytes. For the superficial zone, 5–10 individual cells were randomly chosen and analyzed in each sample. For the middle and deep zone, cells in 5–10 randomly chosen chondrons were analyzed in each sample. Fluorescence intensity in individual cells was normalized with the mean background intensity obtained in three randomly chosen blank areas^42^. The calcium peak magnitude was reported as the mean fold increment of the peak intensity over the mean baseline fluorescence intensity in all tested chondrocytes. The responsive rate was determined by the number of cells whose calcium peak magnitude was over 2-fold of baseline divided by the number of total cells in the superficial zone or each chondron in middle and deep zone. For analyzing the number of peaks during the 15-min recording period, peaks with increment larger than two were counted as one peak, and the peak number was presented as the total number of peaks during the 15-min recording period.

### Histology evaluation

For validating the severity of OA in acquired biopsies, the samples used for calcium imaging were fixed after calcium imaging in 4% paraformaldehyde at 4 °C overnight, followed by decalcification for 1 week with 10% EDTA before embedding in paraffin. Serial sections were cut at up to 5 µm thickness and stained with H&E, alcian blue, and sirius red respectively. Cartilage sections were then evaluated using ICRS grading system as previously reported^43^.

### RNA extraction and RT-qPCR

Articular cartilage for RT-qPCR and Western boltting collected from TKA surgery with informed consent obtained from participant patients. Cartilage samples collected form non-degenerated and early-degenerated regions were processed into small fractions with a tissue homogenizer (Precellys 24, Bertin, MD, USA) at 4 °C. Total RNA was extracted using RNeasy® Mini Kit (Qiagen, Hilden, Germany). The purity of RNA was measured and quantitated on a Nanodrop-1000 spectrophotometer (Thermo Scientific, USA). l μg total RNA was used for cDNA synthesis. GAPDH was used as an internal reference, and target gene primers were presented in the supplementary information (see Supplementary information). RT-qPCR was performed in a reaction volume of 25 μl using QuantiTect SYBR Green PCR kit (Qiagen, Hilden, Germany). Assays were performed in triplicates, and the mRNA levels were normalized to GAPDH using the ∆∆CT method.

### Western boltting

Protein fraction was prepared with RIPA Lysis Buffer (Beyotime, Beijing, China) using tissue homogenizer (Precellys 24, Bertin, MD, USA), and 20 μg/lane of proteins were subjected to SDS-PAGE (10%). The blots were incubated with anti-Ca_v_3.1, Ca_v_3.2, Ca_v_3.3, A2D-1, and β-actin (Abcam, MA, USA), Nfatc2-dephos and Nfatc2-phos (Sigma, MO, USA) antibodies (1:100 dilution) and then incubated with anti-rabbit/mouse horseradish peroxidase-conjugated IgG (Sigma, MO, USA). GDS8000 detection system (UVP, USA) was used for the detection of the bound antibody.

### Statistical analysis

Data were presented as mean ± SEM. For demographic data of patients, the statistical significance of age and BMI was determined by one-way ANOVA with Bonferroni’s post hoc test. For spatiotemporal features of spontaneous [Ca^2+^]_i_ signaling, the statistical significance of the normalized fold increment, the number of peaks, and the responsive rate among all groups were determined by two-way ANOVA with Bonferroni’s post hoc test. Spearman’s rank correlation coefficient method was used to analyze the relationship between each parameter of [Ca^2+^]_i_ signaling to ICRS grading system. For RT-qPCR and Western boltting data, the data from OA groups were compared with a hypothetical value of 1.0 (Normalized Normal control value) by using the student’s t-test. Statistical significance was defined with *p* < 0.05.

### Data availability statement

The datasets generated during and/or analyzed during the current study are available from the corresponding author on reasonable request.

## Electronic supplementary material

Supplementary Table S1

Supplementary Figure S1
