# Supplementary material for: Altered spontaneous calcium signaling of in situ chondrocytes in human osteoarthritic cartilage
Source: Sci Rep. 2017 Dec 6;7:17093. doi: 10.1038/s41598-017-17172-w (PMC5719003; doi:10.1038/s41598-017-17172-w)
Supplement: Supplementary file 1 — Supplementary Table S1 [file 41598_2017_17172_MOESM1_ESM.doc]

**Title:** Altered spontaneous calcium signaling of *in situ* chondrocytes in human osteoarthritic cartilage

**Names of the authors:** Xiaoyuan Gong^1^, Wenbin Xie^1,2^, Bin Wang^3^, Lingchuan Gu^1^, Fuyou Wang^1^, Xiang Ren^1^, Cheng Chen^1*^ and Liu Yang^1*^

1. Center for Joint Surgery, Southwest Hospital, Third Military Medical University, Chongqing 400038, PR China
2. Department of Joint Surgery, First Affiliated Hospital, Hunan Normal University, Changsha, Hunan 410005, PR China
3. Institute of Life Sciences, Chongqing Medical University, Chongqing 400016, PR China

**Grant supporters:**

National Natural Science Foundation of China (31130021, 11602046)

Southwest Hospital, Third Military Medical University (SWH2016JCYB-32)

**Corresponding author’s information:**

**Liu Yang:** 30 Gaotanyan Main St., Shapingba Dist., Chongqing 400038, China, +86-18523029454, [jointsurgery@163.com](mailto:jointsurgery@163.com)

**Cheng Chen:** 30 Gaotanyan Main St., Shapingba Dist., Chongqing 400038, China, +86-13648424986, cclljjff@163.com

| Name | Forward | Reverse |
| --- | --- | --- |
| ACAN | TCGAGGACAGCGAGGCC | TCGAGGGTGTAGCGTGTAGAGA |
| ADAMT-4 | ACTGGTGGTGGCAGATGACA | TCACTGTTAGCAGGTAGCGCTTT |
| ADAMT-5 | GAACATCGACCAACTCTACTCCG | CAATGCCCACCGAACCATCT |
| MMP-1 | ATGTGGACCATGCCATTGAGA | TCAGAGACCTTGGTGAATGTCAGA |
| MMP-3 | CGGTTCCGCCTGTCTCAAG | CGCCAAAAGTGCCTGTCTT |
| MMP-13 | AGCGCTACCTGAGATCATACTACCA | TCATGGAGCTTGCTGCATTCT |
| SOX-9 | GCACTCATAATATGGCATCCTTCA | AAACACGAACACAAACCAAAGCTT |
| SOX-11 | GGTGGATAAGGATTTGGATTCG | GCTCCGGCGTGCAGTAGT |
| COL1A1 | AACGCGTGTCAATCCCTTGT | AGAACGAGGTAGTCTTTCAGCAACA |
| COL2A1 | TGGACGATCAGGCGAAACC | GCTGCGGATGCTCTCAATCT |
| GAPDH | ACGGATTTGGTCGTATTGGG | CGCTCCTGGAAGATGGTGAT |

Supplementary Table S1 RT-qPCR primers design for tested genes
